# Peptide Based Vaccine Approaches for Cancer—A Novel Approach Using a WT-1 Synthetic Long Peptide and the IRX-2 Immunomodulatory Regimen

**DOI:** 10.3390/cancers3043991

**Published:** 2011-10-25

**Authors:** Paul H. Naylor, James E. Egan, Neil L. Berinstein

**Affiliations:** IRX Therapeutics, 140 W 57th Street, New York, NY 10019, USA; E-Mails: pnaylor@irxtherapeutics.com (P.H.N.); jegan@irxtherapeutics.com (J.E.E.)

**Keywords:** IRX-2, synthetic long peptides, Wilms' tumor gene 1, WT-1, cancer vaccine, immunomodulator, adjuvant

## Abstract

Therapeutic cancer vaccines have the potential to generate a long lasting immune response that will destroy tumor cells with specificity and safety, in contrast to many other current cancer therapies. Clinical success to date has been limited by a number of factors including choice of immunogenic cancer rejection antigens, optimization of vaccine platforms and immune adjuvants to effectively polarize the immune response, and incorporation of strategies to reverse cancer mediated immune suppression by utilization of effective adjuvant/immune modulators. WT-1 (Wilms' tumor gene 1) is a cancer antigen that is required for tumorigenesis, expressed in a high percentage of tumor cells and rarely expressed in adult normal cells. Moreover spontaneous immunity to WT-1 is seen in cancer patients and can be augmented with various therapeutic vaccine approaches. IRX-2 is an immune modulator with demonstrated preclinical and clinical pleiotropic immune activities including enhancement of the immune response to potential tumor antigens. This paper presents the rationale and preclinical data for utilizing the WT-1 tumor antigen in a novel vaccine platform consisting of a synthetic long peptide containing multiple class I and class II epitopes in combination with the IRX-2 immunomodulatory regimen to overcome immuno-suppressive pathways and enhance the anti-tumor response.

## 1. Introduction

Cancer vaccines have the potential to generate an immune response that will destroy tumor cells with significantly better specificity and safety than current cancer therapies [1-4]. Unfortunately, clinical success with therapeutic vaccines to date has been modest, in part due to issues related to antigens and adjuvant/immune-modulator selection. An effective therapeutic vaccination approach for cancer needs to address several challenges. Firstly, one must select an antigen that is differentially, yet homogeneously expressed in tumor cells, but not in critical organs or tissues. Ideally the antigen will be important in tumor pathogenesis and thus conserved in proliferating tumor cells. Also, there must be evidence that the immune system has not been completely tolerized to the self-antigen and can respond to it. Secondly, the antigen must be presented to the immune system in an effective vaccine platform that effectively induces cellular responses specifically to the antigen. In order to be effective these responses would need to enhance CD4 and CD8, but not T regulatory responses. Finally, the vaccination approach needs to address the many pathways of tumor induced immune suppression in order to generate truly functional immune anti-tumor responses. Thus, it is not surprising that despite the testing of multiple tumor antigens in clinical trials and evaluating a variety of therapeutic cancer vaccine regimens, there remains an underlying theme of immune dysfunction that will require effective immune modulation and enhancement for therapeutic cancer vaccines to provide clinical efficacy.

### 1.1. Therapeutic Peptide Vaccine Approaches for Cancer

Therapeutic cancer vaccines based on peptide epitopes have been the most widely evaluated due to the simplicity of production, safety, specificity for targeted epitopes and success in preclinical models [5-8]. Initial studies used only class 1 epitopes (∼9 aa) that were HLA restricted and administered with adjuvants. Subsequent studies have improved the responses by using both class I (CTL) and class II (T helper) epitopes along with various adjuvants. Also evaluated with modest success were analogues (mimotope) of peptides that were designed to bind more efficiently to class I receptors than the native peptides. Studies by several groups have reported convincing immune response data for multiple peptide vaccines targeting multiple tumor antigens and multiple MHC. More recently, slightly longer peptides that were shown to contain both class I and class II overlapping peptides within their sequence were shown to generate class I and class II T cell responses. Although these studies have met with minimal success with respect to enhancing survival, they have lead to a developing consensus that longer peptides which contain multiple class I and class II overlapping epitopes have increased potential as effective immunogens in a therapeutic cancer vaccine. The specific studies supporting this hypothesis include those with modest sized peptides targeting Her-2/neu, NY-ESO-1, CEA, WT-1 and HPV [5-8].

### 1.2. Adjuvants for Peptide Vaccines

The critical events for T cell-mediated anti-cancer immune response include antigen presentation to T cells primarily in the lymph nodes draining the source of the antigen (*i.e.*, tumor or immunization site), followed by T cell activation in the lymph nodes and migration of cytotoxic cells to the peripheral sites. Although cytokines play a critical role in effective immune activation, relatively few studies have utilized combinations of cytokines as vaccine adjuvants [3,5,9]. The early vaccine studies were with “classic adjuvants” such as Complete and Incomplete Freund's adjuvants and their derivatives either alone or in combination (MPL, TDM, MDP, bacterial DNA sequences, poly I:C, squalene, mineral oil *etc.*). The results were interpreted as an increase in immune response due to the activation of the “inflammatory” cytokine network which would drive the immune response. With the observation that many components of Freund's adjuvant activated TLR receptors on antigen presenting cells, studies evolved to evaluate purified and/or modified TLR based adjuvants as stimulators of cytokine production via activation of antigen presenting cells. More recently, the use of viral constructs to deliver antigens is believed to stimulate cytokine production via innate immune mechanism since many TLR's are activated by viral products. In the studies where cytokines were used as adjuvants, the cytokine studied most often was GM-CSF administered locally to enhance monocyte precursors. The other was rIL-2 which has been used systemically to increase antigen specific T cell proliferation [3,8,9].

### 1.3. IRX-2

IRX-2 is a primary cell-derived cytokine biologic that enhances immune response and induces tumor rejection in preclinical studies [9-12]. IRX Therapeutics has developed a proprietary cGMP, robust, and scalable process that generates IRX-2 with a demonstrated consistent profile of multiple cytokines [10,11]. The cytokines in IRX-2 have been shown to preferentially enhance the cell-mediated components of the immune response. In preclinical studies, IRX-2 activates dendritic cells as well as T cells. *In vivo* it has been demonstrated to be a potent immune modulator with adjuvant activity [9,12-15] (Table 1).

This enhancement of the immune response occurs due to the presence of multiple immune active cytokines such as IL-1β, IL-2, TNF-α, and IFN-γ. In Phase I and II clinical trials for patients with head and neck squamous cell carcinoma (HNSCC), the IRX-2 immunomodulatory regimen has been reasonably tolerated and resulted in increased lymphocyte infiltration (LI) into the tumor at surgery relative to pre-treatment biopsy, which was associated with increased survival [9,10,15]. Based on the histological assessments, presumably immune responses to endogenous tumor antigens were being generated by the IRX-2 immunomodulatory regimen.

Although cytokines have been evaluated as immune adjuvants, relatively high doses of cytokines (especially rIL-2 and rGM-CSF) have been used; potentially synergistic physiological levels (*i.e.*, ng/mL rather than (μg/mL) of cytokines that target the many different immune cell types have not been evaluated in a therapeutic vaccine setting. While IRX-2 is a complex primary cell produced biologic with consistent ranges of multiple cytokines and chemokines with synergistic activity in preclinical studies, the contribution of individual cytokines in this biologic have not yet been completely defined. The physiologic cytokine levels in IRX-2 are much lower than concentrations of recombinant cytokines used in *ex vivo* DC maturation or in high dose systemic cytokine therapies. The ability of IRX-2 to activate both DCs and T and even B and NK cells makes it especially attractive since it is this combination of immune cell subsets that coordinate the immune response against tumor antigen expressing cells.

### 1.4. WT-1 is an Immunogenic Tumor Antigen in Clinical Evaluation

WT-1 (Wilms' tumor gene 1) is a highly immunogenic protein expressed intracellularly during normal early development, but subsequently downregulated in adults [17-21]. Its over-expression is required for leukemogenesis and proliferation of many solid tumors. Furthermore, the close homology between WT-1 in mice and humans and the identification of T cell epitopes that are identical for both species offers a unique preclinical model for evaluation of vaccine response [18,20-24]. In addition, this homology provides for a useful pre-clinical mouse model using WT-1 expressing murine tumor cells (TRAMP-C2) to evaluate anti-tumor activity with both prophylactic and therapeutic vaccines [22].

Since WT-1 is a potentially useful tumor antigen, a number of clinical trials have evaluated WT-1 peptide based vaccines (Table 2). The trials have been small and were not placebo controlled so while clinical efficacy remains unconfirmed, it can be inferred by comparing responses to those predicted for the stage of the individual patient's disease [25-34]. The two dominant peptides used in the studies were RMFPNAPYL or its analogue YMFPNAPYL which are HLA-2 restricted or CMTWNQMN or its analogue CYTWNQMNL which is an HLA-24 restricted. The primary formulation of the peptide vaccine was oil:water using Montanide as the “adjuvant”. For example, Sugiyama and colleagues pioneered this field with a clinical trial where he and his colleagues treated 26 patients with breast or lung cancer, myelodysplastic syndrome (MDS) or acute myeloid leukemia with three different doses of the HLA-A24 wild type or modified WT-1 peptide in Montanide [32]. There were no serious adverse events described. Severe leukopenia occurred in two patients with MDS. Twelve of 23 evaluable patients had clinical responses and clinical responses correlated with antigen specific tetramer responses. The Sugiyama group also treated 21 patients with HLA-A24 recurrent glioblastoma with intradermal injections of 3 mg of the modified HLA-A24 peptide [31]. The vaccine was well tolerated. Two patients had partial responses and 10 patients had stable disease. Although the frequencies of WT-1 T cell precursors in the glioblastoma patients were higher than that seen in healthy controls, no increases were seen after vaccination in clinical responders or non-responders.

The Heike group evaluated the HL-A2 and HLA-A24 peptides in combination with gemcitabine in Japanese patients with advanced pancreatic or biliary tract cancer [32]. Each patient received the peptide that was optimal for their HLA genotype. The peptide was administered in Montanide with booster immunization every two weeks. T cell responses were assessed using peptide specific tetramers and cells expanded against peptide with IL-2 for 10–14 days. A positive response after 2–12 immunizations was detected in 13/20 patients as compared to 1/25 for pretreatment samples. Immune responses were seen with both the HLA-A2 (8/14) and the HLA-A24 (5/11) peptides.

The Scheinberg group evaluated a four-peptide vaccine which included the nine amino acid analogue of RMF (YMF), two helper epitopes, and a longer peptide containing the YMF peptide and additional sequences predicted to be helper epitopes [27,28]. GM-CSF was administered at the vaccine site as an immunomodulator. The patient population for the two studies included mesothelioma (n = 9), non-small cell lung cancer (n = 3) and patients in remission from acute myeloid leukemia (n = 9). The patients were not restricted to HLA-A2 since a response to helper epitopes was also being assessed. The overall immunogenicity of the vaccine was 14 CD4+ responses out of 20 evaluable patients and eight CD8+ responses out of nine evaluable HLA-A2 patients.

Thus, over all, in the small studies to date, the majority of peptide immunized patients (29/55 = 53%) had a CD8 tetramer positive response when measured after *in vitro* re-stimulation. While these studies confirm the immunogenicity of the peptides, they also suggest that the optimal vaccine delivery system even when helper epitopes were included has not yet been identified.

Although these small peptide based vaccines have proven to target immunogenic epitopes in the WT-1 tumor antigen, the weakness of the immune response suggests they are not yet optimal. Issues that need to be addressed in future vaccine constructs include HLA restriction of small peptides, requirement for helper epitopes, limit of the immune response to dominant epitopes defined in *in vitro* studies and a need for improved adjuvants that can further enhance the T cell response. A possible solution to many of these issues may be the use of synthetic long peptides (SLP) as a vaccine delivery system [35]. These are novel vaccine platforms consisting of 30–40 amino acid peptides bearing binding epitopes for multiple MHC class I and II complexes [36-39]. These SLPs are hypothesized to be more diversely immunogenic against WT-1 relative to conventional class I or II binding peptides, since they may bind multiple MHC types, inducing both helper and CTL responses. SLPs have induced a higher magnitude immune response to a dominant peptide from Human Papilloma Virus (HPV) E7 protein in pre-clinical models compared to the nonamer peptide. Furthermore, SLPs to the HPV E6 and E7 proteins have produced durable objective clinical responses in patients with vulvar intraepithelial neoplasm in over 80% of subjects for up to two years in a Phase II clinical trial [38]. A caveat of these anti-viral studies is that while they have demonstrated superiority of long peptides to short peptides in both human and non-human models, the protein is a “foreign” protein for which tolerance is not predicted. In fact, this specific issue may limit the use of long peptides to P53 since a CD4 T cell response but not a CD8 T cell response was detected in immunized individuals [39]. Whether a more effective adjuvant would enhance the CD8 T cell response to tumor antigens that have greater levels of tolerance remains to be determined. On the other hand, a number of studies also suggest that a positive CD4 T cell response is associated with improved survival [40-41].

### 1.5. Novel Long Peptide Vaccine Approach-WT-1 SLP and IRX2

We have explored a novel vaccine approach utilizing a SLP to WT-1 combined with the IRX-2 immunotherapeutic regimen. We have studied this approach in a murine model in which the human WT-1 protein, because of its high degree of homology to murine WT-1 is seen as a self protein similar to that for human tumors. We selected for these preclinical studies to focus on the HLA-A2 peptide epitope that has been used in several clinical studies because it is recognized by HLA-A2 humans and has also been reported to be immunogenic in C57Bl/6 mice (Table 3). The long peptide was designed with the primary criteria that the amino acid sequence be 30–35 amino acids, that it contains both class I and class II binding epitopes and that it comes from a region of WT-1 that was identical between human and mouse. The sequence has the RMFPNAPYL epitope in the center and contains predicted class II C57Bl/6 binding epitopes. The predicted epitopes are arranged in table 1 by binding scores and the scores are all consistent with strong binding epitopes. In addition to the RMFPNAPYL sequence scoring well in the C57Bl/6 mice and human HLA, it also scores well in Balb/c mice, suggesting it has the potential to be a universally dominant T cell epitope.

Also listed in Table 3 are the sequences within the long peptide that are predicted to be HLA-A2 and HLA-DRB binders. The studies reported here used the C57Bl/6 mice since the TRAMP-2 tumor cells that express high levels of WT-1 are syngeneic in this strain and have been used in a previous study to demonstrate successful anti-tumor immune reactivity using an adenovirus-WT-1 construct [22]. We evaluated the SLP activity in comparison to the short peptide (RMFshort) in the setting of a self-protein where tolerance towards the dominant epitope is presumed to be present to some degree. In addition to comparing immune activity to the predicted dominant peptide either as a short or long peptide, we also evaluated immune activity in long peptide immunized mice to additional peptides selected using a class 1 binding algorithm.

## 2. Methods for Peptide Vaccine-IRX-2 Studies

### 2.1. Peptide Vaccination Studies Using WT-1 Peptides with or without IRX-2

**Figure 1.**
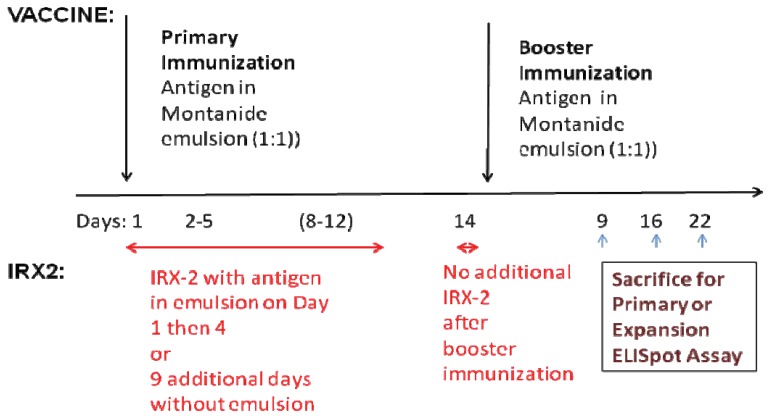
Experimental design for assessing antigen-specific immune response to peptide vaccines in Montanide (IFA) with or without IRX-2. The days of immunization are indicated with the upper arrows and the days of IRX-2 administration with the lower arrow.

C57Bl/6 female mice (6–8 weeks of age) were purchased from either Charles River Laboratories (Wilmington, MA, USA) or Harlan Laboratories (Indianapolis, IN, USA) and were housed under the care of the Cold Spring Harbor Laboratory (CSHL) Animal Facility (Animal Welfare Assurance certificate number A3280-01). All procedures were approved by the Institutional Animal Care and Use Committee of the CSHL. Immunizations were given subcutaneously at the base of the tail to provide for rapid draining to the regional lymph nodes. The WT1 peptides were given at a dose of 10 (μg/mouse. All peptides vaccinations were administered as an emulsion with Montanide (IFA Seppic Inc.) at a 1:1 water to oil ratio. IRX-2 (100 IU/mouse) was incorporated into the emulsion as the aqueous component and administered with the antigen and Montanide subcutaneously on the first day. As indicated in the Figure 1, four or nine additional injections of IRX-2 also at the base of the tail followed the primary immunization but not the booster immunization.

Mice not receiving the IRX-2, had VIVO-10 ™ media substituted as a control containing no cytokines. Booster immunizations with antigen plus adjuvant were given on day 14 but additional daily injections of IRX-2 were not given along with the booster immunizations. Multiple experiments were performed at different times post booster immunization and the data presented is representative of those experiments. Since the ELISpot assays have a variable response on different days, these studies do not demonstrate kinetics of the response as that would require staggered starts for the immunizations and sacrificing of mice at various times post boost on the same day.

### 2.2. ELISpot Assay

ELISpot plates (Millipore, Billerica, MA, USA) were coated with capture antibody (MAB-18 from MabTech (Mariemont, OH, USA) in PBS overnight. Plates were washed, antigen (10 (μg/well) and cells (3 × 10^5^ cells/well) added and then incubated for 18 h at 37 °C. When cells were used directly from the mouse spleen, the protocol was designated as a primary ELISpot assay. After the overnight incubation, wells were vigorously washed with PBS to remove cells and biotinylated second antibody. (MabTech) was added for additional 18 h incubation at 20 °C. Plates were developed using sequential biotin-AP and DAB substrate. Plates were read using a microscope with digital conversion software by ZellNet (Fort Lee, NJ, USA). For some studies, cells were expanded for 6 days using peptide pulsed EL-4 cells for antigen presentation prior to ELISpot (designated as expansion in the figure legends).

### 2.3. Epitope Mapping

Mice that were immunized with the long peptide in Montanide both with and without IRX-2 were used to assess the response to the four predicted T cell epitopes found in the long peptide (see Table 3). For these studies, ELISpot assays were used to define the peptide specific response to the long peptide as well as to the four short peptides. Because the epitope studies required a vigorous response to the long peptide, the ELISpot response of mice from three separate long peptide immunization experiments (regardless of whether they received IRX-2 or not) were combined and then sorted for the strongest response to the long peptide. The difference between the media and the individual predicted epitope peptides was calculated and used to define the breadth of the peptide specific response in long peptide immunized mice.

## 3. Results from Peptide and IRX-2 Vaccine Studies

**Figure 2.**
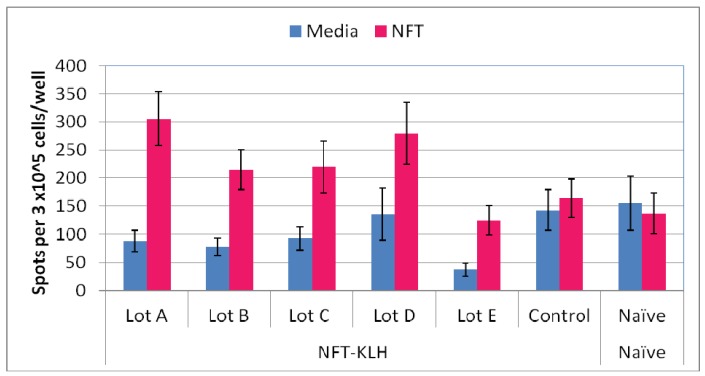
Consistency of IRX-2 bioactivity as defined by enhancement of T cell response in a peptide conjugate vaccine model using the primary ELISpot assay. The mouse dominant epitope from human PSMA (NFT) was conjugated to KLH and used to immunize mice with the protocol described in detail in [12] and outlined in Figure 1. The peptide specific ELISpot assay comparing the baseline response of cells from the mice (media) to the *in vitro* response to incubation with peptide (NFT) was used to evaluate the T cell enhancement following immunization. The data presented are for cells from the lymph nodes of mice which received NFT-KLH and one of five different lots of IRX-2 or a control preparation. The control arm of the study was the NFT-KLH conjugate administered to the mice with a control preparation that consisted of only X-Vivo 10 media that did not contain the cytokines. Also shown is the ELISpot response of lymph node cells from naïve (non-immunized) mice. The results are presented as average ± SEM for 5–7 mice per group for the media *vs.* peptide specific ELISpot response. Also shown in the graph is the increase over media which defines the peptide specific T cell response. All lots of IRX-2 were active (p < 0.05 for media *vs.* peptide as compared to control and naïve for media *vs.* peptide which were not).

IRX-2 enhances the activity of APCs and T cells in both *in vitro* and *in vivo* assays [12-15]. IRX-2 is also an effective adjuvant for various vaccine delivery systems providing the rationale for further studies with a variety of vaccine candidates [9,12]. For example, mice immunized with IRX-2 and the mouse T cell dominant peptide from human PSMA consistently showed increased T cell activity in lymph node and spleen lymphocytes compared to no IRX-2 [12]. This was true for both the peptide administered with Montanide and the peptide conjugated to KLH and given without Montanide. Figure 2 presents data using the PSMA peptide (NFT) conjugated to KLH as the vaccine, administered with any of five different lots of IRX-2. The protocol was similar to that described in our previous paper (and in Figure 1). An enhanced antigen specific T cell response of the lymphocytes from the immunized mice as defined by the ELISpot assay (where the background signal was subtracted) was observed with all lots of IRX-2. The positive antigen specifiic response was compared to both a control preparation consisting of the protein matrix without cytokines and to naïve (non-immunized) mice.

This experiment, in addition to demonstrating the influence of IRX-2 on the T cell response to a vaccine, also used multiple different lots of IRX-2 to demonstrate the manufacturing consistency of IRX-2. In this study the control mice were immunized with the peptide conjugate but instead of IRX-2 they received the media without cytokines. Because the study represents a combination of data over three days of sacrifice, the appropriate statistical assessment is peptide specific response not increase over the non-peptide stimulated naïve or control mice nor increase over the non-peptide response of control or naïve mice. In other experiments, IRX-2 was found to be superior to both CpG and MPL adjuvants, which specifically target Tol-like receptors (TLRs), when the T cell response was assessed using the *in vivo* delayed type hypersensitivity reaction (DTH) assay [12]. With respect to the biological relevance of the IRX-2 enhancement of the T cell response, mice immunized with the NFT-KLH conjugate and IRX-2 controlled the growth of NFT expressing tumor cells more effectively than mice immunized with NFT-KLH and no IRX-2 or KLH alone with or without IRX-2 (40% reduction; p < 0.05 [12]). Since the WT-1 short peptide epitope (RMFshort (RMFPNAPYL), see Table 2) is identical to that of the native protein in the mouse, we demonstrated that there is some level of tolerance to this peptide by comparing the immune response to that for a known foreign and highly immunogenic HPV viral peptide (HPVshort). The predictor score for the HPVshort epitope (RAHYMIVTF) was not as strong as that of the RMFshort peptide (17 *vs.* 24) although the epitope was previously shown to be the dominan tepitope in C57B/6 mice immunized with HPV E7 protein [36]. Both peptides were used as immunogens administered in Montanide either with or without five days treatment with IRX-2 followed by a booster immmunization as described in the material and methods section.

When the spleen cells from the mice were tested in the ELISpot assay for T cell specific responses to the peptides, more mice had a positive immune response to the HPV peptide than the WT-1 peptide (data not shown). The magnitude of the T cell response to the WT-1 short peptide was also weak compared to the HPV peptide and an enhancement with IRX-2 was not readily demonstrated. In contrast, IRX-2 significantly enhanced the peptide specific response to the HPV peptide. We interpret this observation to indicate that immune tolerance to the short WT1 peptides was limiting the antigen specific T cell response since the binding score using the prediction algorithm was in fact higher than that of the HPV peptide. Thus, this was a relevant model to study responses to a self tumor antigen for which tolerance was apparent.

**Figure 3.**
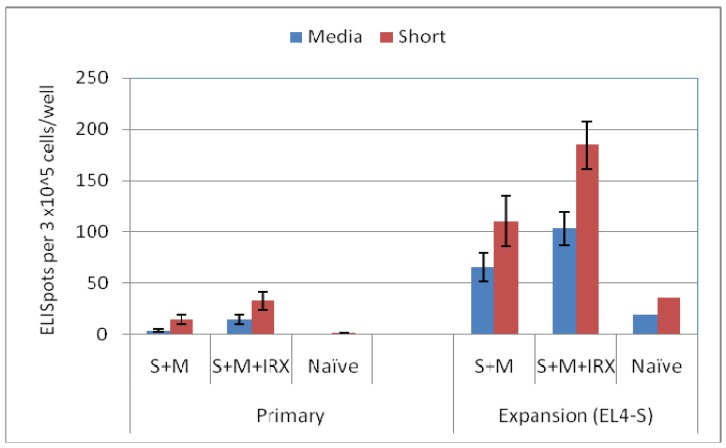
Comparison of primary and expansion ELISpot response of cells from short peptide (S) immunized mice. Mice were immunized with the RMFshort peptide in Montanide (S+M) with or without IRX-2 as indicated in the x-axis. Spleen cells from mice harvested at nine days after a booster immunization were either used directly in the ELISpot (primary assay) or expanded for six days with IL-2 and irradiated EL-4 cells pulsed with the RMFshort peptide [expansion (EL4-S)]. Peptide specific T cell response of the cells from immunized mice was assessed using the RMFshort peptide as *in vitro* stimulating antigen and compared to the non peptide containing media. The results for the expansion assay are presented as ELISpots using the spleen cells incubated with the RMFshort peptide pulsed EL-4 cells. Spleen cells incubated with irradiated EL-4 cells that were not pulsed with peptide had equal or fewer spots, consistent with peptide specific expansion in the presence of IL-2 (data not shown).

Since the immune response of the short peptide immunized mice was weak, cells from immunized mice were expanded *in vitro* by incubation with antigen presenting cells and IL-2 for 6 days before measuring the T cell response to the short peptide. As shown in Figure 3, expansions using peptide pulsed EL-4 irradiated cells, were effective in the short peptide immunized mice when compared to the low primary ELIspot response without *in vitro* expansion. Thus when the expansion protocol was used, it was possible to demonstrate that mice immunized with the short peptide with IRX-2 had a stronger T cell response than mice receiving only the short peptide in Montanide.

**Figure 4.**
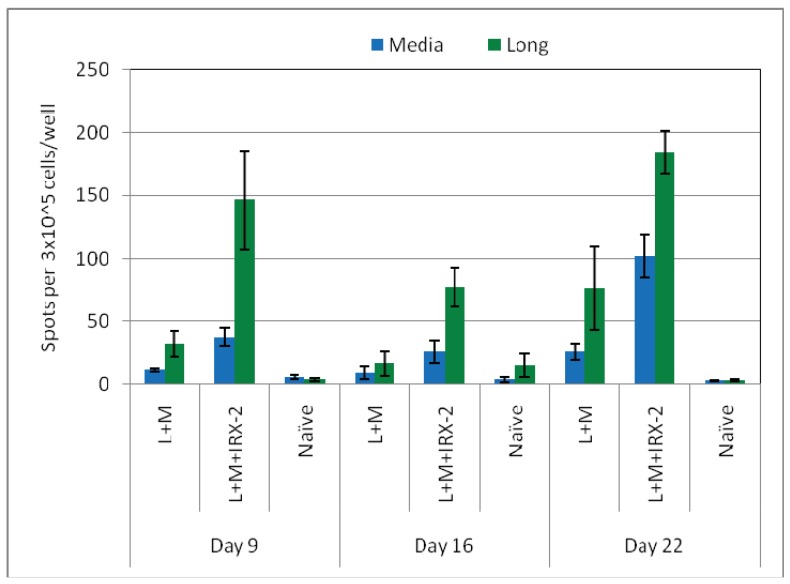
Peptide specific primary ELISpot response of RMFlong (L) peptide immunized mice from separate experiments sacrificed at the time indicated after the booster immunization. Mice were immunized with the RMFlong peptide (L) in Montanide (L+M) with or without IRX-2 as indicated in the x-axis. Spleen cells from the mice at the days indicated after the booster immunization were used in the ELISpot and the specific T cell response was assessed using the RMFlong peptide as *in vitro* stimulating antigens. Results are presented as mean ± standard error of the mean for 5–7 mice per group and are representative of multiple studies performed at various times post immunization. The data does not represent a formal kinetic experiment since the ELISpot assays were performed on different days with different sets of mice. Only the splenocytes from the long peptide immunized mice who received IRX-2 had long peptide specific T cells greater than the media control at all three times after a booster immunization (p < 0.05).

The long and short RMF peptides have been evaluated for immunogenicity in multiple experiments performed at different times after booster immunization (Figures 3 and 4, and data not shown). In all cases, IRX-2 enhanced the T cell response, consistent with our previously published observations (12).

**Figure 5.**
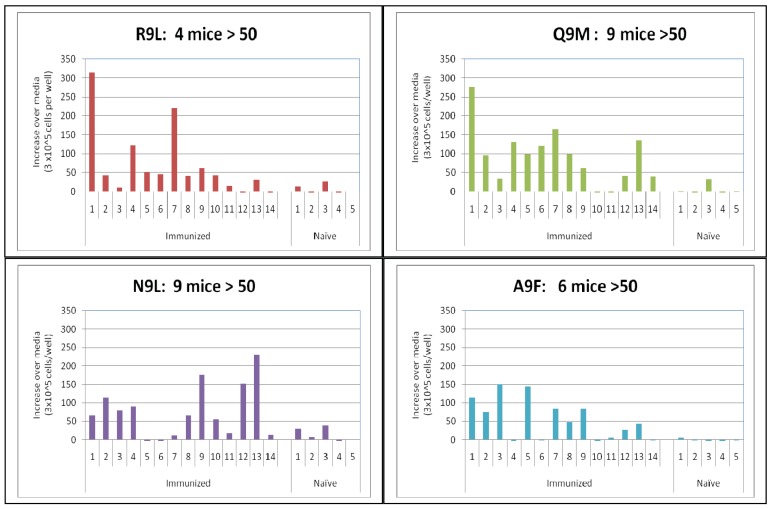
Epitope specific primary ELISpot response of long peptide immunized mice. Spleen cells from mice immunized with the long peptide were evaluated for peptide specific ELISpot response 9–16 days after booster immunization. The peptides evaluated in the ELISpot included the long peptide and four of the peptides representing epitopes predicted to be positive in the C57Bl/6 mice (see Table 2). The results are presented for mice selected from three separate experiments based on the presence of a strong ELISpot response to the long peptide regardless of whether they received IRX-2 or not in order to evaluate the contribution of the individual epitopes to the response. naïve mice from the three experiments are included as negative controls. The mice are numbered sequentially and ranked based on the magnitude of the increase over the media control for the long peptide. The results shown are for the epitope specific increase and values greater than 50 spots (2 × average of naïve mice) are defined as positive. See table 4 for comparison of response of mice immunized with the RMFlong peptide and the HLA predicted binding scores.

To assess whether the mice immunized with the long peptide recognized multiple epitopes, the four best scoring CTL/CD8 binding epitopes in the long peptide (Table 2) were used as target epitopes in the ELISpot assay. As shown in Figure 5 and Table 4, mice that were selected because they were positive for the long peptide (regardless of whether they received IRX2 or not) recognized multiple epitopes whether defined by the number of mice with an ELISpot response greater than media (*i.e.*, % positive mice) or ranked by magnitude of the response (*i.e.*, greater than 2 × the naïve mouse controls; >50 ELISpots). While the response rate ranged from 28%–64% for the individual peptides, the overall response rate as defined by a response to at least one of the peptides was 86%

## 4. Discussion

The advantages of peptide-based vaccines include safety, specificity and ease of cGMP production. However there are also limitations with small peptide vaccines including MHC restriction, lack of helper activity and weak presentation of antigen by APC due to exchange into the MHC as compared to cross presentation processing. In addition only limited clinical anti-tumor activity has been documented to date with these types of peptide vaccines. While the limitations can be somewhat overcome by using multiple peptide vaccines which include both class I and class II epitopes, the processing issue can only be overcome by larger constructs such as recombinant protein or longer peptides with overlapping MHC I and II epitopes. In fact, processing and expression of both class I and class II epitopes on the same APC is considered to be the goal of an effective vaccine. Recombinant proteins, while they may solve the limited epitope dilemma, have proven difficult to make in large quantities under cGMP specifications and the processing of the large proteins may be less efficient than for shorter peptides. Synthetic long peptides (SLPs) represent novel and yet currently underutilized vaccine constructs. They are easy to produce and also require processing for presentation. The limited preclinical and clinical studies with SLP's have proven that they are safe, effective and have the potential to generate immune responses to multiple epitopes. Dramatic clinical regressions have also been seen with one SLP vaccine targeting HPV in patients with vulvar intraepithelial neoplasia. Our studies have provided additional evidence that long peptides to a tumor antigen to which there is immune tolerance are immunogenic and may have immunologic advantages over short peptides (target multiple MHC epitopes).

We have also shown that the antigen-specific T cell responses to the WT-1 SLP are further increased by combining the vaccine with IRX-2. IRX-2 is a multi-targeted primary cell-derived biologic that shapes the adaptive immune response toward effective T cell activation. The synergistic activity of the cytokines in IRX-2 is directed towards both the antigen presenting cells and the activated T cells resulting in a novel T cell immune modulator. Preclinical studies have demonstrated IRX-2 anti-tumor activity in tumor models especially in conjunction with cyclophosphamide and chemo-radiation. Enhancement of the response of mice to several vaccine constructs has also been demonstrated. With respect to mechanism of action of IRX-2, among the many immune activities demonstrated for IRX-2 *in vitro* are activation of antigen presenting cells (dendritic, monocytes/macrophages and Langerhans cells) and T cells. While the T cell activity of cytokines such as IL-1, IL-2 and IFN-γ are well defined, only recently has the activity on APCs been appreciated. In addition to increasing the activation of DCs as measured by surface expression of many activation markers, IRX-2 reversed the depressed antigen processing activity of dendritic cells from cancer patients [15]. In addition to influencing T cell proliferation and differentiation, IRX-2 has also been shown to reverse tumor induced death of T cells thus providing an additional mechanism of action for IRX-2 in a tumor vaccine model [14].

Combining a peptide vaccine that contains multiple class I and class II epitopes (*i.e.*, an SLP) with a synergistic cytokine adjuvant/immunomodulator (IRX-2) in a protocol designed to reduce tumor mediated suppression of the immune response should lead to an improved and more immunogenic therapeutic tumor vaccine with enhanced anti-tumor activity. Both clinical and preclinical studies with IRX-2 support the hypothesis that IRX-2 enhances the immune response to tumor antigens and generates immune mediated anti-tumor activity. As described in this manuscript, the use of IRX-2 and synthetic long peptides (SLPs) should overcome many of the limitations of the peptide tumor vaccine studies to date. Clinical evaluation of this novel combination is in the planning stages.

## 5. Conclusions

We have developed a novel immunotherapeutic approach in attempt to enhance the immunogenicity and clinical activity of peptide-based therapeutic cancer vaccines. We focused on the Wilm's Tumor 1 (WT1) tumor associated antigen. To target multiple MHC types, induce CD4 as well as CD8 responses and enhance antigen specific immunogenicity we have generated a synthetic long peptide (SLP) to WT1. We have also combined this vaccine with the IRX-2 immunomodulator that has been shown in various models to enhance multiple arms of the immune response and increase the immunogenicity of various therapeutic cancer vaccines. Our pre-clinical results show that vaccination with the WT1 SLP induces a T cell response to multiple embedded class I binding epitopes and that the magnitude of the T cell responses is increased by immunomodulation with IRX-2. These results support further evaluation of this novel therapeutic cancer vaccine approach in the clinic.

## Figures and Tables

**Table 1.** Immunologic effects of IRX-2.

**Cells**	**Effects**	**References**
Antigen Presenting Cells	Increase cell surface markers (MHC1, MHCII, CD83, CD40, CD54, CCR and co-stimulatory molecules) Increases morphological appearance of activation Functional effects	13,15
T Lymphocytes	Proliferation and Differentiation Protects T cells from tumor-induced cell death Increase in T cell infiltration of tumors and regional lymph nodes	9,14,16
NK cells	Increases NK and ADCC activity *in vitro*	*
B cells	Increases B cell infiltration of tumors	16
Cancer Vaccines	Increases antigen specific T cell responses to antigens	12

* Unpublished observations from T Whiteside and F Farzin.

**Table 2.** Early phase clinical trials with WT-1 based peptide vaccines.

**Primary Investigator** (Date; Reference) **Population** (Number of patients)	**Immunogen**	**Adjuvant(s) Immunizations** [wks]	**Immune** **Assays**	**Comments** (T cell responses typically measured after *in vitro* restimulation)
Heike (2011; [32]) Pancreatic/Billiary (25)	RMFPNAPYL CYTWNQMNL	Montanide (with gemcitabine) [0,2,4,6,8]	CD8 (Tetramer) DTH to WT-1 peptide	2/25 DTH positive; 13/22 Tetramer positive
Keilholz(2011; [26]) AML/MDS (19)	RMFPNAPYL	GM-CSF (−3,−2, −1,0,+1) KLH (0) [0,2,4,6,8]	CD8 (Tetramer and IFN-γ/TNF-α)	CD8(8/18); 9/13 Stable Disease
Scheinberg (2010; [27]) Acute Myeloid Leukemia (9)	4 peptides: YMFPNAPY; LSGQAYMFPNA PYLPSCLES; RSDELVRHHNM HQRNMTKL; PGCNKRYFKLS HLQMHSRKHTG	Montanide GM-CSF (-2 and 0) [0,4,6,8,10,12 (+6)]	DTH CD4 (proliferation) CD8 ELISpot/Tetramer	DTH (2/9); CD4 (7/9); CD8(3/9); 5/9 stable patients
Scheinberg (2010; [28]) Lung Cancer (12)	4 peptides: YMFPNAPY; LSGQAYMFPNA PYLPSCLES; RSDELVRHHNM HQRNMTKL; PGCNKRYFKLS HLQMHSRKHTG	Montanide GM-CSF (-2 and 0) [0,4,6,8,10,12 (+6)]	DTH CD4 (proliferations) CD8 ELISpot/Tetramer	DTH (3/9); CD4 (6/9); CD8(5/6)
Rezvani (2008; [20]) AML/CML/MDS(8)	PR1 and WT-1 (RMFPNAPYL) peptides	Montanide GM-CSF (0) [0 (single dose)]	CD8 (Tetramer and IC IFN-γ) (PBMC not expanded prior to assay)	Tetramer: 2/8 pre *vs.* 5/8 post to WT-1 2/8 pre *vs.* 7/8 pos to PR-1; 8/8 to either antigen
Sugiyama (2008; [31]) Glioblastoma (21)	CYTWNQMNL	Montanide [weekly]	CD8(Tetramer)	Tetramer positive pre with no increase
Sugiyama (2004; [32]) BC/LC/Leuk (26)	CMTWNQMNY or CYTWNQMNL	Montanide [0,2,4]	CD8 (Tetramer and IC IFN-γ)	12/23 Tetramer Positive after vaccination
Sugiyama (2006; [33]) solid tumors (10)	CYTWNQMNL	Montanide [0,2,4,6,8]	no immune monitoring	NA

**Table 3.** Binding predictions for epitopes found in long peptides.

**RMF_long_ (SLP)**	PFGPPPSQASSGQA**RMFPNAPYL**PS**C**LESQP		
**Epitope (Class 1)**	**C57Bl/6** *	**Balb/c** *	**HLA**
**RMFPNAPYL**	24	17	22 (A02), 19 (B27)
NAPYLPSCL	17	12	***
QASSGQARM	15	9	
ASSGQARMF	12	19	13 (A03)
PYLPSCCLES	3	19	13 (A24)
ASSGQARMF			15 (A11)
FPNAPYLPS			17 (B07)
**Epitope (Class II)**	**C57Bl/6** **	**Balb/c** **	**HLA-DR** *
QARMFPNAPYLLPSC	0.675	.485	22 (DRB1-01) 18 (DRB1-15)
PSQASSGQARMFPNA	0.453	.490	
FPNAPYLPSCLESQP	0.414	.282	
PFGPPPSQASSGQAR	0.326		
GQASSGQARMFPNAP		.278	.
SSGQARMFPNAPYLP			20(DRB1-01)
ARMFPNAPYLPSCLE			22(DRB1-04) 16(DRB1-07) 16 DRB1-11)

* Binding scores for Class I peptides using algorithm available online at www.syfpeithi.de [42];

** Binding scores for class II peptides (I-Ab and I-Ad respectively) using algorithm MHC2Pred available online at www.imtech.res.in/raghava/mhc2pred [43];

*** No score indicates that the peptide was not found in the highest four scores.

**Table 4.** Predicted HLA peptide binding score and immune response in mice immunized with the long peptide. See Figure 5 for response of individual mice and relationship between responses of individual mice to each peptide.

**Sequence**	**Score**	**Number > 50 spots**

RMFPNAPYL (R9L)	22	4/14 = 28%
NAPYLPSCL (N9L)	17	9/14 = 64%
QASSGQARM (Q9M)	15	9/14= 64%
ASSGQARMF (A9F)	12	6/14 = 43%
Positive for one or more	NA	12/14 = 86%
